# Comparing the Efficacy, Safety, and Superiority of Calcitonin Gene-Related Peptide Monoclonal Antibodies and Botox in Preventing and Treating Migraines

**DOI:** 10.7759/cureus.13002

**Published:** 2021-01-30

**Authors:** Mariah Siddiqui, Parth V Shah, Prachi Balani, Angel R Lopez, Chelsea Mae N Nobleza, Safeera Khan

**Affiliations:** 1 Neurology, St. George's University, True Blue, GRD; 2 Neurology, California Institute of Behavioral Neurosciences & Psychology, Fairfield, USA; 3 Medicine, California Institute of Behavioral Neurosciences & Psychology, Fairfield, USA; 4 Internal Medicine, California Institute of Behavioral Neurosciences & Psychology, Fairfield, USA; 5 Psychiatry, California Institute of Behavioral Neurosciences & Psychology, Fairfield, USA

**Keywords:** migraines, cgrp, botox injections, headache disorders, calcitonin gene-related peptide, migraine with aura, side effects of medical treatment

## Abstract

Both calcitonin gene-related peptide (CGRP) monoclonal antibodies (mAbs) and OnabotulinumtoxinA (botox) are used in the prevention of chronic migraines. However, it is not clear which is more effective overall. This review will compare the efficacy, side effects, cost-effectiveness, and other factors between CGRP mAbs and botox. We searched Pubmed and Google Scholar using the keywords migraines, CGRP mAbs, botox, efficacy, side effects, aura. All articles, including case-control/cohort studies, case series, case reports, randomized control trials, traditional/systematic reviews, were analyzed. CGRP mAbs and botox both reduce the frequency of migraines in patients. Patients have reported they decreased migraines' frequency and intensity in several studies after being given each medication. While CGRP mAbs are more recent medications, botox has been studied for more than a decade as a migraine preventative. Both drugs have minor short-term side effects, but some CGRP mAbs may cause persistent constipation too. CGRP mAbs are self-injected every month, and botox is physician-injected every three months, making it easier to stay compliant. While both medications are expensive, botox has a lower cost over time. Botox is more effective prophylaxis of migraines based on the articles that were reviewed. While both CGRP mAbs and botox are efficacious and tolerable, botox has been studied longer, has fewer side effects, is more cost-effective, and is easier to comply with.

## Introduction and background

Studies show that 15%-17% of women and 6% of men suffer from migraines [1]. A migraine is defined by a severe pulsing or throbbing sensation in the head unilaterally but may also occur bilaterally. It is a neurovascular headache disorder which may be multifactorial, recurrent, and debilitating. Usually, increased intracranial pressure and an aura are present before or during a migraine. Auras include increased sensitivity to light, sound, smell, and some migraines may even be associated with vomiting and nausea [2]. These prodrome symptoms can be due to several triggers such as alcohol, lack of eating, scents such as perfumes, hormones, stress, and weather [3]. While the exact cause of migraines is unknown, there is evidence that different brain pathways might be involved in migraines' pain. For example, some data suggest that brain excitability and the trigeminovascular system's sensitization is modified in certain individuals due to congenital mechanisms [4]. The auras associated with the migraines might also be due to activation of the trigeminal afferents leading to certain phenomena that induce pain [5]. Certain biomarkers are also thought to play a role in migraine pain. Serotonin, glutamate, GABA, and dopamine were all found to be involved in the nociceptive signaling of migraine pain, suggesting a balance of activating and suppressive activity taking place [6]. Studies also show that migraines may even cause referred pain to the body's external areas, such as the periorbital skin [7].

Chronic migraines are defined as having 15 or more migraines per month for at least three months. Many potential chronic migraine treatments have been studied for the past few years. Calcitonin gene-related peptide (CGRP) is thought to play an essential role in both preventing and treating migraines [8]. CGRP is a neuropeptide that is released from sensory nerves and participates in mechanisms related to pain. One study showed that those given CGRP had more intense headaches amongst migraine patients than those who weren't [9]. CGRP is made in the neurons and vasodilates blood vessels during migraines, making it a target for migraine therapy. Therefore, CGRP monoclonal antibodies (mAbs) were studied and used to block the release of CGRP by antagonizing its receptor and limiting its effects on the nociceptive response. In a randomized trial with 1672 participants, it was reported that patients who received a CGRP mAbs had fewer migraines than those who received placebos [10]. While CGRP mAbs are expensive and have some small side effects, they are both efficacious and tolerable, making them promising drugs for migraine patients [11].

Another promising treatment and preventative for chronic migraines is botox. Derived from Clostridium botulinum, this toxin causes paralysis of muscles and is thought to block the pain sensation associated with migraines. Botulinum toxin is injected by a physician into the pericranial muscles in patients every three months [12]. Botox is thought to cause its effects due to inhibiting neurotransmitter release from primary sensory neurons [13]. This allows the toxin to suppress the pain, which may be due to excessive muscle contractions, which lead to migraines [14].

While data show that both CGRP mAbs and botox effectively treat and prevent migraines, there is not much information that compares the effectiveness between the two or discusses which is a more potent preventative. This article will compare and discuss the efficacy of using CGRP mAbs versus botox and comment on a more powerful drug. It will also compare other factors such as side effects, costs, and patient compliance to analyze which one is more favorable.

## Review

Method

Pubmed and Google Scholar were the databases that we used to search for the articles. The articles were found and analyzed in September and October of 2020. The keywords used were: migraines, CGRP mAbs, botox, efficacy, side effects, and aura. The articles were screened by reviewing articles with titles and abstract that were relevant to the topic being discussed.

Inclusion Criteria: The inclusion criteria were articles between 1990 and 2020, articles in English, articles on humans and animals, abstract, or full text.

Exclusion Criteria: The exclusion criteria were grey literature. 

Results

A total of 40,513 articles were found using the keywords, and after applying the criteria listed above, it was shortened to 9,255 articles. Twenty-nine articles that were relevant to our review were used in this article using the criteria mentioned above, along with choosing articles that analyzed the efficacy of CGRP mAbs and botox in regards to migraines. Of the 29 articles, there were 13 review articles, two narratives, three animal studies, three human clinical trials, four randomized controlled trials (RCTs), and five observational studies. The articles' main points discussed the efficacy of CGRP mAbs or botox for migraines, the mechanisms of actions of the two drugs, the typical side effects of the drugs, and the benefits and drawbacks of each drug.

Mechanism of action

Migraines are headaches that can be unilateral, bilateral, present with an aura, and be accompanied by nausea and vomiting. Usually, non-steroidal anti-inflammatory drugs (NSAIDs) are given to terminate the pain. However, a few drugs are being used to prevent migraines from occurring in the first place. Many of the current medications used are off-label, such as beta-blockers and anti-psychotics, and not intended for migraines. CGRP mAbs and botulinum toxin are two recent interventions that seem to be more promising in preventing chronic migraines. Three CGRP mAbs, galcanezumab, erenumab, and fremanezumab, were approved by the Food and Drug Administration (FDA) to treat migraines in 2018. Botox was approved by the FDA in 2010 for migraine treatment and has been studied longer to prevent migraines.

**Figure 1:**
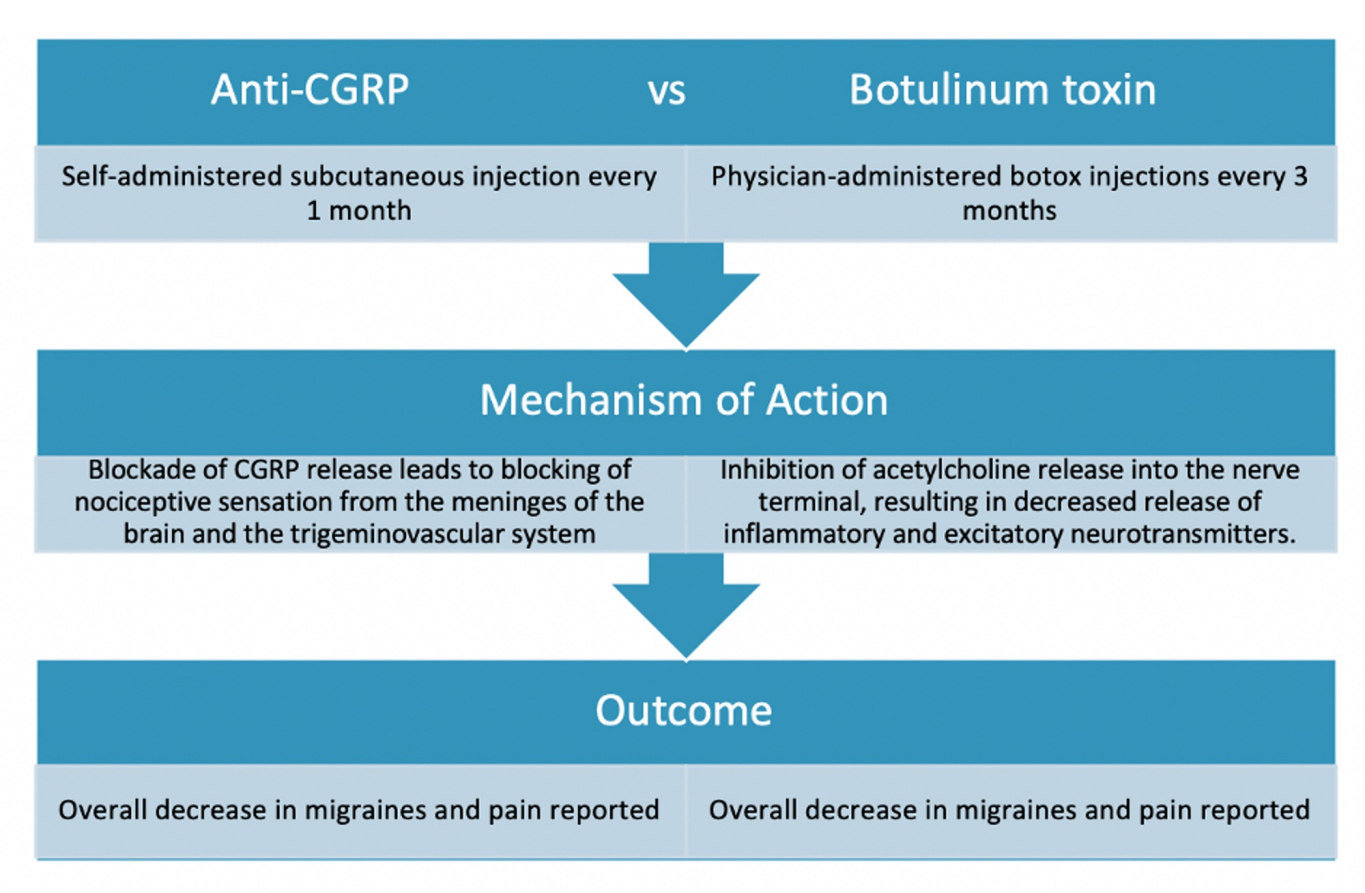
Difference in the mechanism of action between CGRP mAb drugs and botulinum toxin. CGRP: calcitonin gene-related peptide; botox: OnabotulinumtoxinA; mAb: monoclonal antibody.

CGRP mAbs and botulinum toxin A work differently to help prevent migraines, as shown in Table 1 and Figure 1. Migraines may be caused by multiple mechanisms that are not fully known. One such pathway is the trigeminovascular system, which modulates sensation and pain in the face and head. It was discovered that CGRP is one of the main neurotransmitters involved in migraines [15]. CGRP is a neuropeptide that allows signaling in the trigeminovascular pathway and allows cranial blood flow and pain transmission to occur, leading to migraines [16]. CGRP causes inflammation and irritation of the meninges, leading to migraines. This is how pain occurs; this is also a pathway that can be blocked to prevent migraines.

**Table 1:** Studies showing the mechanisms of action of CGRP mAbs and botox. CGRP: calcitonin gene-related peptide; mAbs: monoclonal antibodies; botox: OnabotulinumtoxinA.

Author and year of pub	Drug studied	Type of study	Results	Conclusion
Russo et al. 2015 [15]	CGRP mAbs	Review	CGRP is a vital neurotransmitter that plays a role in migraines.	Antagonists and mAbs against CGRP can be helpful in preventing migraines.
Moriarty et al., 2019 [16]	CGRP mAbs	Review	CGRP plays a main role in the vasodilation and sensation of trigeminovascular system.	CGRP mAbs are useful in preventing migraines and have many advantages.
Cavestro et al., 2019 [17]	CGRP mAbs	Review	Specific neurotransmitters such as CGRP are thought to play a role in migraines through the trigeminovascular system.	Monoclonal antibodies against CGRP can be used as a treatment for migraines.
Yuan et al., 2020 [18]	Botox	Review	Botox disrupts neuropeptide secretion that is related to trigeminal signaling, preventing the sensation of pain.	Botox is safe and efficacious in preventing chronic migraines.
Lew et al., 2002 [14]	Botox	Review	Botox inhibits acetylcholine release, leading to local paralysis and blockade of pain.	Botox can help with pain relief via its mechanism of action.
Escher et al., 2017 [19]	Botox	Review	Botox is a migraine prophylactic that might work by inhibiting acetylcholine release and CGRP release.	Botox can inhibit migraine pain via blocking acetylcholine release.
Szok et al., 2015 [20]	Botox	Review	155-195 units of botox were found to be effective in patients.	Botox was effective in preventing migraines in patients.

Monoclonal antibodies have been created to block CGRP and be used as prophylactics [17]. The monoclonal antibodies, such as galcanezumab (Emgality) or Erenumab, work by blocking either the CGRP ligand or receptor. They are monthly self-injectable drugs that come with a subcutaneous auto-injector the patients can do themselves. Another migraine prophylactic, botulinum toxin, works using a different mechanism. Clostridium botulinum is a bacterium from which the neurotoxin, botulinum toxin, is derived. Botox is typically used in cosmetics to diminish wrinkles' appearance since it relaxes the face's smooth muscles. Botox is also indicated as a preventive medicine given for migraines to those who have more than 15 migraines a month. While the full process is unknown, it is thought that botox works by altering the secretion of neuropeptides in the trigeminal pain pathway [18]. It does this by inhibiting acetylcholine release from the neuromuscular junction, which induces paralysis [14]. Some studies show that botox can also affect the release of CGRP and other neurotransmitters relating to pain [19]. Around 155-195 units of botox is injected into 31 areas on the face, head, neck, and shoulders [20]. Patients must get the injections every 12 weeks, and it takes a few weeks for the pain-relieving effect of botox to start. 

Benefits of CGRP mAbs vs. botox

CGRP mAbs have several benefits of being used as prophylactic migraine treatments, as shown in Table 2. They are proven to be very productive in several trials. As shown in the table above, a clinical trial split 1,672 patients randomly into groups getting different doses of galcanezumab and a placebo group. The patients were divided into groups with a 120mg dose, a 240mg dose, and a placebo in a 1:1:2 ratio. In the group given 240mg, 60.9% of the patients stated they had at least a 50% reduction in their headaches.

**Table 2:** Studies comparing the benefits of CGRP mAbs vs. botox. CGRP: calcitonin gene-related peptide; mAbs: monoclonal antibodies; botox: OnabotulinumtoxinA.

Author and year of pub	Drug studied	Type of the study	Results
Do et al., 2019 [21]	CGRP mAb	Review	One thousand six hundred seventy-two patients were split randomly into groups of different dosages of galcanezumab and a placebo. 60.9% of the patients on 240 mg showed a >50% decrease in migraines. CGRP mAb was proven to decrease migraines and not have any detrimental side effects.
Akhtar et al, 2019 [11]	CGRP mAb	Review	Monoclonal antibodies against CGRP are much safer than their predecessors. There aren't any major short-term side effects.
Deen et al., 2017 [22]	CGRP mAb	Review	Monoclonal abs against CGRPs are more efficacious and have fewer side effects than older migraine-prophylaxis. They also don't have drug-drug interactions and do not have any significant hepatotoxic side effects. It is also given only once a month instead of daily, increasing compliance.
Yalinay et all, 2018 [23]	Botox	Observational	Two hundred forty-five patients enrolled in the study, and 180 answered questions about their treatment experience. 82.9% of patients felt that botox helped control their headaches. On a scale of 0-10, a mean score of 6.94 ± 2.4 was given for the effectiveness.
Dodick et al., 2010 [24]	Botox	Randomized control trial	A total of 1384 patients were given either a placebo or botox. Those on botox reported a more considerable decrease in migraines than those on the placebo
Chan et al., 2009 [25]	Botox	Observational	Twelve adolescents from ages 14-18 were given botox injections. All six long-term patients stated they experienced a better quality of life and had decreased migraines.

Additionally, in the group given 120mg, 62.3% reported the same [21]. It was also shown that there were not any major short-term side effects from using the CGRP mAbs, unlike their predecessors, gepants, which caused hepatotoxicity [11]. A review of the efficacy of monoclonal abs against CGRP showed that the drugs were efficacious and well-tolerated in many patients in many trials. Additionally, since monoclonal antibodies are metabolized into amino acids and peptides, they don't interact with any other drugs, making it easier for people to take multiple medications [22]. These specific monoclonal antibodies against CGRP are taken only once a month, whereas many of the previous prophylactic medications were daily. This allows patients to remain compliant with their medications.

Botox also has several benefits as opposed to other prophylactic medications. A cohort study done in 2018 followed 245 patients with chronic migraines who were given botox for several cycles. One hundred eighty of the patients remained in the trial, and 82.8% of those patients reported they felt their migraines were more controlled with the botox [23]. A more extensive randomized, double-blind study was done in 2010 with 1,384 patients, where 688 patients were given botox, and 696 patients were given a placebo. There was a significant decrease in the frequency of headaches in the patients who were given botox.

Moreover, since this procedure is done every 12 weeks, it was found that it was easier to be compliant. There were also no major long-term side effects noted aside from the usual effects of botox [24]. A study was done in adolescents, which showed that botox was effective and safe to use in younger patients. Since migraines can start at any age, some individuals experience them around a young age close to puberty. In a group of six patients between the ages of 14-18 on whom other migraine preventatives didn't work, botox was given during a clinical trial. It was found that every six patients had a decrease in their headaches and did not suffer from any major side effects [25]. 

Drawbacks of CGRP mAbs vs. botox

While both CGRP mAbs and botox are effective in different ways in migraines, they also have some downsides, as shown in Table 3. CGRP mAbs need to be self-injected by the patient every month, and it was found to have some side effects, such as causing a rash and pruritus at the injection site [11]. It is also an expensive drug and can cost up to $600 without insurance every month, making it less accessible to some people. The injection itself is also quite painful since it is injected into the subcutaneous tissue. The median flow rate of one of the medications, Emgality (galcanezumab), is 0.4mL/s over four seconds [26]. This may cause pain since a liquid is dispersing into the muscle at a fast rate. Another RCT showed that in a trial with 483 patients, some common side effects included upper respiratory tract symptoms, along with fatigue and headaches [27]. For certain CGRP mAbs, constipation was also a major side effect in more than 20% of patients in a retrospective study in 2020 [28].

**Table 3:** Studies comparing the drawbacks of CGRP mAbs vs. botox. CGRP: calcitonin gene-related peptide; mAbs: monoclonal antibodies; botox: OnabotulinumtoxinA; BMI: body mass index.

Author and year of pub	Drug studied	Type of study	Results
Akhtar et al. 2019 [11]	CGRP mAb	Review	Monoclonal antibodies against CGRP are expensive and can cause a rash for a short time after injection.
Dou et al., 2020 [26]	CGRP mAb	Observational	The CGRP mAb, Emgality, has a fast flow rate of injection.
Sun et al., 2016 [27]	CGRP mAb	Randomized controlled trial	Most common adverse effect was fatigue, headache, upper respiratory problems.
Robbins et al., 2020 [28]	CGRP mAb	Observational	A retrospective study from October 2018 to January 2020 evaluated 119 patients who took a CGRP mAb. Some monoclonal antibodies such as erenumab caused constipation in more than 20% of the patients.
Dodick et al., 2010 [24]	Botox	Randomized control trial	Adverse effects include neck pain, muscular weakness. Resolves typically after a while.
Yalinay et all, 2018 [23]	Botox	Observational	Two hundred forty-five patients enrolled in the study, and 180 answered questions about their treatment experience. 82.9% of patients felt that botox helped control their headaches. However, of the 245 patients, only 31 were treated for 12 months, showing that botox compliance may be low. Also, this study's yearly cost claimed it was for one year of botox was $3000.
Wu-Fienburn et al., 2018 [29]	Botox	Experimental	Based on the BMI of the patients, botox had a different effect on migraine prophylaxis.

Botox also has several disadvantages that may affect patient compliance. In an RCT done on 1384 adults, it was found that there were more side effects present in those patients with botox as opposed to the placebo group. Some of these side effects include muscular weakness and neck pain. Eyelid ptosis was also noted among many of the patients in this study [24]. While this does resolve after a while and is expected from a medication like botox, it is still an important factor to note since it can decrease patient compliance. Even though botox is done every three months, the total cost of botox treatment for migraines can cost around $3,000, making it very hard for many people to afford, thus decreasing compliance [23]. Another drawback of botox is that its effectiveness may be affected by the BMI of the patient. An experimental study done in 2018 showed that botox was not as productive in patients with a higher BMI. This could be because there is more distance between the skin where botox is injected and the muscles that it targets typically [29]. This might limit who can utilize botox for their chronic migraines.

Limitations

One of the limitations of this review is not having information about long-term side effects for either medication. Also, there is no study currently that has published results that compares the efficacy of the two drugs in a clinical trial. There is no study or data on whether or not the efficacy would increase if given both CGRP mAbs and botox simultaneously to prevent migraines, which would be an interesting question to pursue.

## Conclusions

CGRP mAbs and botox are effective as preventatives in many studies. While they both work in different ways, they seem to have a similar impact of inhibiting the nociceptive pathways involved in headaches. While CGRP mAbs are thought to inhibit the sensation of pain by antagonizing a major inflammation marker such as CGRP, botox does it by inhibiting the release of acetylcholine in neuromuscular junctions. Recent studies that tested the efficacy of CGRP mAbs and had a large sample size proved that these drugs were potent preventatives for chronic migraines. Botox was shown to be effective in multiple studies over the past decade. Regarding side effects, both CGRP mAbs and botox have mild short-time adverse effects; however, one long-term side effect seen in patients who took CGRP mAbs was constipation. The injections for CGRP mAbs are also much more painful compared to the injections of botox. Another factor to consider when comparing these two medications is cost-effectiveness. While certain insurances partially/fully cover both of the medications, those without insurance might not be able to afford either medication. Botox is the overall cheaper drug long-term, and this could be an option that can be considered for those who have financial restraints. Based on the articles reviewed, it can be said that botox is a more effective, cheaper, and painless option for chronic migraines compared to CGRP mAbs.
