# Correction: Comparing the Efficacy, Safety, and Superiority of Calcitonin Gene-Related Peptide Monoclonal Antibodies and Botox in Preventing and Treating Migraines

**DOI:** 10.7759/cureus.c41

**Published:** 2021-02-12

**Authors:** Mariah Siddiqui, Parth V Shah, Prachi Balani, Angel R Lopez, Chelsea Mae N Nobleza, Safeera Khan

**Affiliations:** 1 Neurology, St. George's University, True Blue, GRD; 2 Neurology, California Institute of Behavioral Neurosciences & Psychology, Fairfield, USA; 3 Medicine, California Institute of Behavioral Neurosciences & Psychology, Fairfield, USA; 4 Internal Medicine, California Institute of Behavioral Neurosciences & Psychology, Fairfield, USA; 5 Psychiatry, California Institute of Behavioral Neurosciences & Psychology, Fairfield, USA

The following statement in table 3 has been corrected as it erroneously stated that the product label has a black box warning for constipation.

"A retrospective study from October 2018 to January 2020 evaluated 119 patients who took a CGRP mAb. Some monoclonal antibodies such as erenumab caused constipation in more than 20% of the patients and now carries a black box warning."

This error was the result of mistakenly citing an older version of the article on a health care provider website rather than the up-to-date version on the journal website. As a result, the statement has been amended to the following and reference 28 has been updated to link to the journal website.

"A retrospective study from October 2018 to January 2020 evaluated 119 patients who took a CGRP mAb. Some monoclonal antibodies such as erenumab caused constipation in more than 20% of the patients."

